# CNV Detection from Exome Sequencing Data in Routine Diagnostics of Rare Genetic Disorders: Opportunities and Limitations

**DOI:** 10.3390/genes12091427

**Published:** 2021-09-16

**Authors:** Beryl Royer-Bertrand, Katarina Cisarova, Florence Niel-Butschi, Laureane Mittaz-Crettol, Heidi Fodstad, Andrea Superti-Furga

**Affiliations:** Division of Genetic Medicine, Lausanne University Hospital (CHUV), University of Lausanne, 1011 Lausanne, Switzerland; katarina.cisarova@chuv.ch (K.C.); florence.niel@chuv.ch (F.N.-B.); laureane.mittaz-crettol@chuv.ch (L.M.-C.); heidi.fodstad@chuv.ch (H.F.); asuperti@unil.ch (A.S.-F.)

**Keywords:** arrayCGH (aCGH), copy number variations (CNVs), exome sequencing (ES), MLPA, next-generation sequencing (NGS), rare and undiagnosed disease, structural variation (SV)

## Abstract

To assess the potential of detecting copy number variations (CNVs) directly from exome sequencing (ES) data in diagnostic settings, we developed a CNV-detection pipeline based on ExomeDepth software and applied it to ES data of 450 individuals. Initially, only CNVs affecting genes in the requested diagnostic gene panels were scored and tested against arrayCGH results. Pathogenic CNVs were detected in 18 individuals. Most detected CNVs were larger than 400 kb (11/18), but three individuals had small CNVs impacting one or a few exons only and were thus not detectable by arrayCGH. Conversely, two pathogenic CNVs were initially missed, as they impacted genes not included in the original gene panel analysed, and a third one was missed as it was in a poorly covered region. The overall combined diagnostic rate (SNVs + CNVs) in our cohort was 36%, with wide differences between clinical domains. We conclude that (1) the ES-based CNV pipeline detects efficiently large and small pathogenic CNVs, (2) the detection of CNV relies on uniformity of sequencing and good coverage, and (3) in patients who remain unsolved by the gene panel analysis, CNV analysis should be extended to all captured genes, as diagnostically relevant CNVs may occur everywhere in the genome.

## 1. Introduction

In clinical practice, the process of finding a molecular genetic diagnosis for rare genetic disorders is challenging. In spite of advances in laboratory technology in the last 10 years, approximately one-half to two-thirds of patients remain without a clear diagnosis, depending on their clinical manifestation [1,2,3]. Establishing a precise diagnosis is the first and important step to help the patient and his/her family, even in the absence of a specific therapy [4].

Human genetic disorders may arise from genetic variations that range in size from a whole chromosome down to a single-nucleotide variant (SNV). In between, a significant proportion of pathogenic variants is represented by sub-microscopic deletions/duplications of ∼50 nucleotides to thousands of nucleotides, that are collectively called copy number variations (CNVs). To detect CNVs, specific techniques such as genome-wide CNV detection (arrayCGH (aCGH)) or locus CNV detection (multiplex ligation-dependent probe amplification (MLPA)) are needed. They can detect CNVs either at a large scale (50 kb) or at the level of a single exon [5]. Optical genome mapping is a novel method allowing the detection with high accuracy of structural variants, particularly CNVs [6,7].

In routine diagnostics, there is no “perfect” genetic technology capable of the simultaneous detection of structural rearrangements, CNVs, SNVs, short-repeat expansions, etc. For this reason, multiple parallel or sequential investigations are often necessary. Guidelines have been set up for different clinical disease groups and diagnostic entities, suggesting specific diagnostic workflows that consider the feasibility, the costs, the conditions of reimbursement set by medical insurances, and the diagnostic yield [8,9].

For many conditions, such as congenital malformation syndromes or developmental disorders, it remains disputed whether the first-tier diagnostic tests should be a next-generation sequencing (NGS)-based gene panel or a microarray-based chromosome analyses (arrayCGH), which is less expensive but has a lower diagnostic yield [10].

The genetic analyses of rare Mendelian disorders have tremendously changed over the past decades. Most of the technologies have evolved towards faster and cheaper analyses with a higher resolution scale. This is particularly the case for NGS. Sequencing of the entire coding regions of the patient’s genome—exome sequencing (ES)—has greatly progressed with libraries targeting exons of all genes simultaneously and with improved coverage and uniformity. These advancements, in combination with bioinformatics software improvements, have allowed direct CNV detection from ES raw data to become more and more precise [11,12,13]. Based on the coverage (reads-depth) of one patient compared to a set of patients sequenced at the same time and/or sequenced using the same conditions, NGS-based CNV software can detect regions with significantly more reads (gain of copies) or fewer reads (loss of copies) than the average [14].

Here, we implemented the detection and validation of pathogenic CNVs using ES data. We used ExomeDepth software [15] with default settings and subsequently parametrised and annotated the CNV results with an in-house bioinformatics pipeline to be used for ES-based CNV analysis in routine diagnostics. We applied our pipeline to 450 NGS patients sequenced between the years 2018 and 2020.

## 2. Materials and Methods

### 2.1. Patients Cohort

Patients recruited in this cohort were part of the diagnostic NGS routine, seen in the Genetic Laboratory of the Genetic Medical Service, at CHUV, Lausanne, between 2018 and 2020. DNA of the patients was extracted after appropriate informed consent, from whole blood for all postnatal cases (*n* = 440) and from the skin (*n* = 2), trophoblast (*n* = 1), or cultivated amniotic liquid (*n* = 7) for prenatal cases, with Blood DNA Kit on Maxwell^®^ 16 or Maxwell^®^ RSC. The median age of the postnatal patients was 22 years (ranging from 1 to 81 years), 237 were males and 203 were females. The ES-based CNVs pipeline was performed on ongoing diagnostic cases in parallel with the SNVs pipeline.

### 2.2. Exome and Targeted-Exome Sequencing

Exome sequencing was performed after obtaining patients’ appropriate informed consent using the SureSelect V7 exome kit from Agilent (Agilent Technologies, Santa Clara, CA, USA) on an Illumina HiSeq 2500 at the Genomic Technology Facility of the University of Lausanne. Targeted exome sequencing was carried out upon patients’ appropriate informed consent using the TruSight One Expended (TsoE) kit from Illumina in our genetic laboratory on an Illumina NextSeq 500. The raw NGS reads were aligned to the human reference genome GRCh37 using Novoalign from Novocraft (http://www.novocraft.com, accessed on 5 August 2017). The data cleanup, followed by variant calling, was performed according to GATK Best Practices recommendations (https://gatk.broadinstitute.org/, accessed on 5 August 2016) as already described in [16]. SNVs were annotated using Annovar [17] in combination with in-house developed scripts. Following the Swiss Society of Medical Genetics (SSMG) guidelines (https://sgmg.ch/, accessed on 5 August 2015), NGS analysis in routine diagnostics is only targeting genetic alterations impacting genes clinically relevant to the patients’ phenotype. Thus, only SNVs impacting the requested genes’ panel were analysed, and they were subsequently filtered according to their quality, rarity, and impact on genes. SNV’s final classification was carried out according to ACMG criteria [18].

### 2.3. Conventional CNV-Detection Methods—Microarray-Based Chromosome Analyses

Genome-wide CNV detection was performed using the Agilent microarray platforms (Agilent Technologies, Santa Clara, CA, USA): (1) Agilent SurePrint G3 Human CGH Microarray (4 × 180 K array) with an overall median probe spacing of 13 kb (11 kb in Refseq genes) and (2) Agilent SurePrint G3 Human (1 × 1 M High-Resolution Microarray) with an overall median probe spacing of 2.6 kb. Microarray processing was carried out according to the manufacturers’ recommendations. The arrays were scanned using an Agilent Microarray Scanner (Agilent Technologies, Santa Clara, CA, USA) and analysed using Agilent Genomics Workbench Lite 6.5 software. In routine diagnostics, a hit is considered significant when a minimum of three probes are deviated consecutively. The global diagnostic resolution for a 180 K array is situated between 60 and 100 kb.

### 2.4. Conventional Locus CNV-Detection Method—MLPA

Locus-specific CNV detection was achieved using the following gene-specific MLPA probe mixes from MCR Holland (MCR Holland, Amsterdam, the Netherlands): P330-PCDH19 (*PCDH19* gene), P033-CMT1 (*PMP22* gene), P165-HSP (*SPAST* gene), and P461 DIS (*STRC* gene). MLPA analyses were carried out according to the manufacturers’ recommendations and analysed using Coffalyser software (MCR Holland). In routine diagnostics, a hit is considered significant when a minimum of two probes are deviated consecutively. If a single probe is deviated, a second method is necessary to exclude a false-positive result due to an allelic dropout, often caused by the presence of an SNV in the probe-specific sequence (MCR-Holland).

### 2.5. NGS-Based CNV Calling, Annotation, and Filtering

CNV calling was performed using the ExomeDepth software [15] with default settings, per batch of patients sequenced at the same time on the same machine following the same bioinformatics procedures. For ES data, a batch is represented by 32 patients, while by 8 patients for the targeted exome TsoE.

For each library’s target, the number of reads expected to be present in each patient was computed, based on the patient coverage as well as of the entire batch’s coverage, and it was compared to the observed number of reads actually present in the sequenced data of each patient [15]. Regions with statistically fewer reads in a patient, compared to the rest of the batch, show a potential loss of copies, while regions that are statistically more covered represent a potential gain of copies. The difference in the coverage level help in assessing the ploidy of the CNV.

CNVs were annotated with an in-house pipeline, summarised in Table S1. The annotations contain the cytogenetic location of the CNVs and the distance from the beginning (pTer) or end (qTer) of the chromosome based on the number of library targets in those regions. The impacted genes were annotated with different information, including the RefGenes name and the number of exons per gene impacted by the CNV, the associated disease description from OMIM if existing (https://omim.org/, accessed on 11 September 2021), the presence or absence of a pseudogene, the DOMINO score [19] and their haploinsufficiency and triplosensitivity using ClinGen Dosage Sensitivity Map (https://www.ncbi.nlm.nih.gov/projects/dbvar/clingen/, accessed on 5 August 2020). The CNV annotation calculates the number of targets present in 180 K and 1 M arrayCGH libraries inside each CNV’s boundaries. CNVs were merged together if they were distanced less than 3 targets away and with the same type of variation (gain or loss of copies) and ploidy, to avoid potential splitting of CNVs due to lower coverage in some targets. The frequency of each CNV was calculated in the batch of patients processed together and in an in-house database of CNVs containing results from previous batches.

Similar to SNVs analysis and in accordance with the Swiss Society of Medical Genetics guidelines, CNVs were filtered based on their overlap with genes present in the clinical panel(s) requested by the patient’s geneticists and/or clinicians.

### 2.6. Pathogenic CNVs

CNVs detected by the above-described pipeline were considered likely pathogenic or pathogenic if they were having the following features: (1) rare or absent from the in-house database of CNVs and in control populations (gnomAD SVs v2.1 [20]; Database of Genomic Variants build GRCh37; nstd102 Clinical Structural Variants (formerly ISCA)); (2) impacting gene(s) associated with diseases similar to the clinical description of the patient; (3) impacting a gene or a region harbouring similar reported pathogenic CNVs (DECIPHER [21]; Human Gene Mutation Database^®^), and according to Nowakowska CNV review [22], as well as ACMG and ClinGen recommendation [23]. For critical genes included in the detected CNVs, ClinGen Dosage Sensitivity Map was used to assess the haploinsufficiency and triplosensitivity of the different genes, as well as gnomAD gene metrics [20]. This is particularly important for the classification of unknown CNVs.

### 2.7. Samples Selection

In the first phase, we selected 5 patients with known pathogenic CNVs previously detected by arrayCGH or MLPA (Table S2), and we sequenced their exomes in a batch with 27 other undiagnosed patients. After processing the entire batch with ExomeDepth software, we only analysed the CNVs detected in those 5 patients in order to develop and parametrise the NGS-based CNV pipeline. In the second phase, we applied this pipeline to all patients to whom ES was performed in 2018 and 2019 (*n* = 199). All potentially pathogenic CNVs detected were then validated with arrayCGH/MLPA (Table 1, Table S3), and the pipeline was fine-tuned at each new discovery. Finally, in the third phase, we applied the NGS-based CNV pipeline to all our NGS patients sequenced in 2020, including both ES (*n* = 93) and targeted-exome (*n* = 158) libraries (Figure S1). No validation was required for CNVs detected at this step, unless atypical CNVs, such as quadruplication, were found. However, most CNVs were confirmed later on by arrayCGH/MLPA for familial segregation and genetic counselling (Table 1). Notably, before the inclusion of TsoE library kit in Phase 3, we tested the conformity with the ES library kit by comparing the SNV and CNV results between 10 samples sequenced with both libraries. The conformity tests were repeated annually with at least 4 samples to ensure the consistency of the results.

## 3. Results

### 3.1. Disease Categories

**Figure 1:**
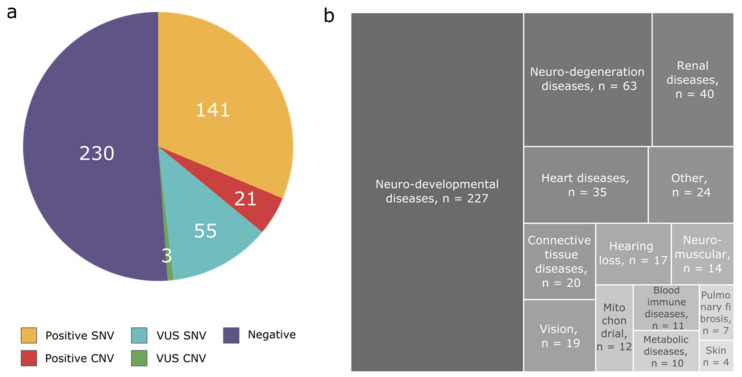
(**a**) Overall diagnostic yield in the 450 patients of the cohort–patient positive with compound heterozygous SNV and CNV affecting the same gene (*n* = 1) is included in the category “Positive CNV” and (**b**) details of the disease categories for the patients.

The samples in this study were part of our routine diagnostic NGS analysis. Of the 450 patients processed with the NGS-based CNV pipeline, approximately half had a neurodevelopmental condition (*n* = 227), 63 had neurodegeneration disorder, 40 suffered from renal disease, 35 from cardiac problems, 20 had connective tissues disease, 19 had vision problems, 17 suffered from hearing loss, 20 patients had a diverse set of diseases, and the remaining categories had fewer number of patients (Figure 1b). Notably, some of these patients had multisystemic diseases and had, therefore, multiple clinical gene panels analysed simultaneously. The majority of patients had only one category of disease requested (*n* = 398, 88.4%), but 2 (*n* = 41, 9.1%) or 3 or more (*n* = 11, 2.4%) categories per patient were also analysed.

### 3.2. Diagnostic Yield

Of the 450 NGS individuals analysed for both SNV- and CNV-impacting genes in their requested clinical genes panels, we detected 162 individuals with diagnostic changes (SNVs and/or CNVs), giving an overall positive diagnostic rate of 36.0% (Figure 1a). A total of 230 cases remained negative, 58 patients had variants classified as a variant of unknown significance (VUS) in their molecular report, caused by SNVs only (*n* = 55) or by CNVs (*n* = 3).

**Figure 2:**
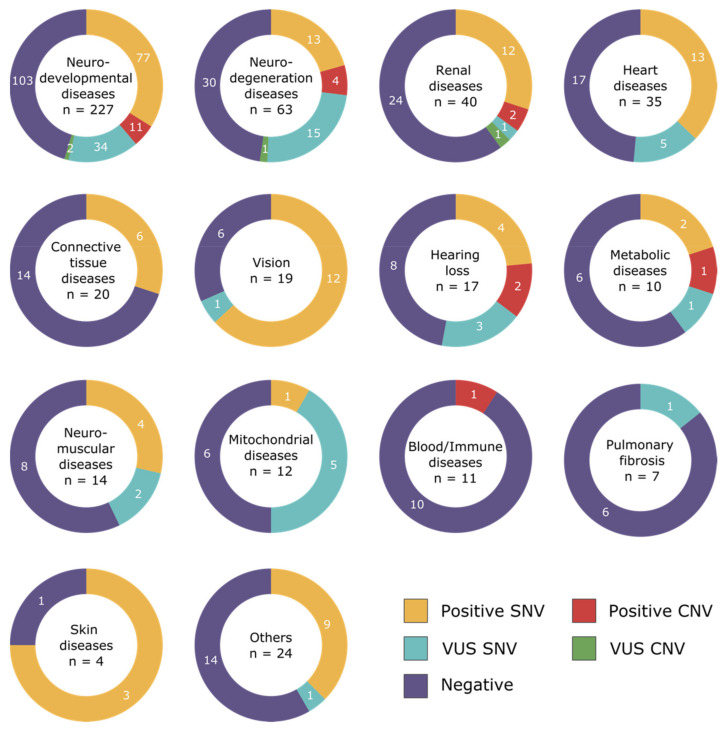
Diagnostic yield per disease category (some patients have multiple categories analysed),—patient positive with compound heterozygous SNV and CNV affecting the same gene (*n* = 1) is included in the category “Positive CNV”.

The positive rate was very variable between disease categories. For categories with more than 10 patients analysed, the highest diagnostic yield was in the vision panel, where a diagnostic result was found in more than 63.2% of the patients (*n* = 12/19). The positive rate was at least 30% in neurodevelopmental disorders (*n* = 88/227, 38.8%), in heart diseases (*n* = 13/35, 37.1%), in hearing loss (*n* = 6/17, 35.3%), in renal diseases (*n* = 14/40, 35.0%), in connective tissues diseases (*n* = 6/20, 30.0%), and in metabolic disorders (*n* = 3/10, 30.0%). The other categories—neuromuscular diseases (*n* = 4/14, 28.6%), neurodegeneration disorders (*n* = 17/63, 27.0%), suspected blood and immune system disease (*n* = 1/11, 9.1%), and mitochondrial disorders (*n* = 1/12, 8.3%)—had less than a third of their cases solved. No clear positive cases were found in pulmonary fibrosis cases (*n* = 0/7, 0%) (Figure 2).

In total, the NGS-based CNV analysis allowed us to detect likely pathogenic or pathogenic CNVs explaining the phenotype of 18 patients, 11 patients were processed during phase 2 of the project, and 7 patients during phase 3 (Table 1, Table S3). The use of the CNV pipeline improved the diagnostic yield by 5.9% for all categories. (Figure 1a). Notably, one patient (case 12) was found to harbour two pathogenic *STRC* variants, both an SNV and a CNV. CNVs classified as VUS were also found in three patients (Table S4). The small number of patients per disease category does not allow us to infer relevant increases in the diagnostic yield for each category separately. The NGS-based CNVs were found in patients suffering from neurodevelopmental disorders (*n* = 11/227), neurodegeneration diseases (*n* = 4/63), hearing loss (*n* = 2/17), renal disorders (*n* = 2/40), metabolic diseases (*n* = 1/10), and blood and immune system disease (*n* = 1/11) (Figure 2). For some categories of diseases, no pathogenic CNVs were found in our cohort (heart disease, neuromuscular diseases, vision, connective tissues diseases, pulmonary fibrosis, and mitochondrial disorders).

### 3.3. Pathogenic CNVs per Disease Categories

The majority of pathogenic or likely pathogenic CNVs detected here were larger than 400 kb (*n* = 11/18, 61.1%)—cut-off used in prenatal arrayCGH testing—and were impacting multiple genes (Table S3). These CNVs were associated with neurodevelopmental phenotype (*n* = 8/11, 72.7%), neurodegenerative disease (*n* = 2/11, 18.2%), or renal disease (*n* = 1/11, 9.1%). They were all known deletions or duplications, detectable by various molecular and cytogenetic methods (Table 1). We also detected gene-size CNVs in four patients (*n* = 4/18, 22.2%). The last three patients (*n* = 3/18, 16.7%) had small CNVs of one or two exons in size. In total, CNVs in two patients would not have been detected by arrayCGH or MLPA, either because of their small size leading to an absence of arrayCGH targets or because of the unavailability of an MLPA kit targeting these exons (cases 3 and 13). The *STRC* heterozygous deletion in case 12 would not have been seen as well by arrayCGH, as there are few arrayCGH targets for the *STRC* gene due to the presence of a pseudogene [25]. However, few exons of *STRC* are targeted by MLPA, including some present in the heterozygous deletion harbored by the patient, which allowed us to confirm the CNV detected in our patient.

### 3.4. Comparison of arrayCGH and NGS CNV Results

In our cohort of 450 NGS patients, 120 had arrayCGH analysis conducted either before the NGS analysis (*n* = 87), during the NGS analysis to validate any interesting CNV (*n* = 15), or after the NGS analysis was found negative (*n* = 18).

Amongst the 87 negative arrayCGH cases performed before the NGS analysis, we reached a molecular diagnosis in 34 patients (39.1%), mostly due to pathogenic SNVs (*n* = 33/34, 97%). One patient referred because of global developmental delay and abnormal social behaviour (case 11) had a 1q21.1 microduplication originally annotated as a VUS in the arrayCGH results from 2013. The same microduplication was found during the NGS analysis, and the literature published after the original evaluation allowed this CNV to be reclassified as pathogenic [26,27,28] (Table 1 and Table S3). From the negative NGS cases, 18 patients had an arrayCGH analysis performed after the NGS analysis. Amongst them, three patients (*n* = 3/18, 16.7%) were found to have a pathogenic CNV explaining the patient’s phenotype and undetected by the NGS-based CNV pipeline (Table 2). These three cases are detailed in the next paragraph.

### 3.5. Pathogenic CNVs Undetected by the NGS Pipeline 

For patient 19, suffering from lissencephaly, SNVs and CNVs applied to a neurodevelopmental panel of 1462 genes came back negative. Full-genome arrayCGH analysis was requested later on, and a heterozygous deletion of the UTR region until the intron 2 of *PAFAH1B1* was found in the patient. Mutations in this gene have been associated with lissencephaly and subcortical laminar heterotopia, inherited in an autosomal dominant manner (MIM:601545, [29]). Interestingly, *PAFAH1B1* was present in the NGS clinical panel analysed in our patient, but the deletion had not been detected by the CNV pipeline. Indeed, the first exon of *PAFAH1B1* was outside the open reading frame of the gene, and it was not targeted by the NGS library used in the patient. The second exon of the gene was present in the ES library, but it was not highly covered in general, even in patients with the two normal copies of the gene. Manual inspection through the ExomeDepth raw data of the patient’s batch showed a reduction in the average read coverage of exon 2 in our patient, compared to other patients sequenced similarly, but the overall difference was too small for ExomeDepth software to consider this single exon to harbour a heterozygous loss of copy (Figure S2).

Patients 20 and 21 both had a negative NGS analysis targeting genes associated with neurodevelopmental disorders. Full-genome arrayCGH analysis was undertaken in a second step, and they both had a pathogenic deletion impacting *NBEA* and *PACS2*, respectively. Variants in the *NBEA* gene have been described in 23 patients with neurodevelopmental disorder with or without early onset generalised epilepsy (NEDEGE; MIM:619157) by Mulhern et al. in 2018 [30], but the gene has not been registered as a Morbid gene on OMIM until January 2021. *PACS2* is also a gene described in 2018 by Olson et al. [31] as being causative of neonatal-onset developmental and/or epileptic encephalopathy, facial dysmorphism, and cerebellar dysgenesis, and similar to *NBEA* gene, it was not included at the time of the analysis in the clinical panel requested for the analysis of patients NGS data. 

### 3.6. Variants of Unknown Significance in arrayCGH Results

In the arrayCGH results, 24 patients had CNVs classified as VUS, with the arrayCGH analysis conducted before the NGS analysis for most cases (*n* = 22/24, 91.7%). These CNVs were further investigated in the NGS results, and 19 of them were detected as well. From the five CNVs not detected, four were in regions without any NGS target, mostly large intronic or intergenic regions. The last undetected CNV, located in the coding region of the genome, was a polymorphic duplication, seen in the control population (gnomAD) and in other patients of the batch.

Globally, the CNVs found both in arrayCGH analysis and with the NGS pipeline are very similar in terms of the number of copies and in size with small differences at the borders of the CNVs (Table 1 and Table S2). The boundaries of the detected CNVs depend on the targets present in the library used, both for arrayCGH and in NGS; the exact breakpoint is in general not known. Thus, the size of the CNVs detected by arrayCGH and the NGS pipeline corresponds to the minimal size of the event.

Interestingly, one case had a duplication in 1q23.3 present in mosaicism, and it was seen both in the arrayCGH results (50–55% of mosaicism) and in the NGS results (58% of mosaicism) (Table S4). The CNV was, however, classified as a VUS since it did not explain the phenotype of the patient.

## 4. Discussion

The gold standard tools for CNV detection in diagnostic settings are currently MLPA and arrayCGH analyses. The main advantage of an arrayCGH analysis is its genome-wide resolution, allowing for the discovery of large gains and/or loss of DNA copies independently of any gene panel. On the other hand, arrayCGH technology is not targeting small CNV events involving one or a few exons [32]. Indeed, we observed that arrayCGH would have missed such small variations in cases 3, 13, and 17, as there were not enough arrayCGH targets around each of those CNVs to ensure their presence with good quality. Those small CNVs are the focus of MLPA analyses, which can be developed specifically for exons of genes known to be often affected by deletions/duplications, such as *PKD1* or *PKD2*, causative of polycystic kidney disease [33]. However, some disorders including neurodegenerative or neurodevelopmental disorders have a large genetic heterogeneity, making the development of targeted MLPA for all the exons of all these genes unfeasible. Additionally, neither arrayCGH nor MLPA can screen for the presence of SNVs.

Exome sequencing overcomes some of these difficulties. First of all, it allows for simultaneous detection of both SNVs and CNVs, thus eliminating the need of using multiple different technologies in one patient, speeding up the diagnostic process. It also permits the identification of large and small CNVs at once (as previously detected by arrayCGH and MLPA, respectively). One of the diseases in which in our study we observed the highest increase in diagnostic yield owing to the CNV pipeline was hearing loss. Pathogenic variants in *STRC* are the second most frequent cause of hearing loss [34,35]. However, a high homology of more than 99% of the coding regions between *STRC* and its pSTRC pseudogene makes this gene difficult to study either by MLPA or Sanger sequencing, as it is very difficult to design appropriate MLPA targets and/or Sanger primers [25]. ArrayCGH is also not the best fitting validation method for the small deletions in *STRC* due to the paucity of arrayCGH targets in the *STRC* region (commercially available arrayCGH provides only one probe in the *STRC* region). NGS-based approaches have already been described as a viable and competitive alternative to classical methods for the detection of CNVs impacting *STRC* [36,37]. Moreover, in our experience with SNVs and CNVs detected in our *STRC*-positive patients, NGS with high quality of coverage (average coverage >100×) and proper library design was superior to all other techniques. In general, genes and regions of genes with high homology (e.g., *SMN1, SMN2, HBA1, HBA2, IKBKG*) will be difficult to target and to sequence, and the CNV ES-pipeline will not be efficient to detect CNVs impacting those regions [38].

In spite of its clear advantages, ES-based CNV detection is no silver bullet and comes with some limitations. Different factors affect the efficiency of ES-based CNV detection. First, batches of samples processed in a similar fashion are needed for the analysis. Second, the coverage of sequencing data needs to be homogeneous within and among samples, to allow differentiation between potential gain or loss of copies, and the technical variability of sequencing. The sequencing coverage is particularly important to distinguish true heterozygous deletions from low-covered regions [39,40]. This is a known issue particularly for the first exons of genes and for genes and exons in low-mappability regions [41]. As sequencing libraries and capture kits are constantly being improved, this issue may become less problematic in the next years [42]. In our laboratory, we added extra steps in the library preparation protocols with a verification of the patients’ DNA quality (TapeStation^®^ from Agilent) and DNA quantity (Qubit^®^ from Invitrogen by Life Technologies and TapeStation) to ensure a maximum uniformity when the patients’ DNAs were pooled together before sequencing.

A further limiting factor of ES-based CNV detection is that only CNVs impacting well-covered coding regions of genes present in the sequenced library will be detected. Intergenic and intronic CNVs are, by default, not visible with the ES-based CNV pipeline, as seen in our study when comparing the NGS and arrayCGH results. The CNVs boundaries detected by the ES-based CNV pipeline are, therefore, to be taken with caution, as most CNVs will likely start outside of the coding regions. Similarly, the breakpoints located in low-covered regions might also pass undetected. 

In the current study, the ES-based CNV analysis was originally based on gene panels, as the SNVs analysis, and only variations impacting genes present in the requested gene panels were analysed. Given that more than 200 disease-associated genes are being discovered each year [43,44], it is challenging to keep the disease gene panels up to date, leading to CNVs being passed over, as seen in patients 20 and 21 (Table 2). In comparison, arrayCGH analysis is performed genome wide, and all CNVs impacting any gene would be examined. Yet, if the gene was not linked to a disease at the time of the analysis, the CNV would be at most classified as a VUS, as seen in case 11. Two options might be envisioned to allow for the detection of CNVs localised outside of the original gene panel. The CNV analysis could be opened to all the coding regions of the genome, similar to the genome-wide arrayCGH analysis, but this means an additional patient’s consent for such analysis will be needed (at least in our institution and following the national Swiss SSMG guidelines). The second option could be the frequent reanalysis of CNVs data, similarly to the reanalysis of SNVs, which has shown great improvement in the diagnostic yield in recent years [45,46,47,48]. It is however a time-consuming task, and its reimbursement by medical insurances may be difficult to obtain.

Two of our cases highlight the possibility that CNVs may be the consequence of unsuspected chromosomal rearrangements. Thus, one might consider the necessity of obtaining additional studies (such as a karyotype or a FISH analysis), particularly for CNVs affecting genes localised in the pTer or qTer regions of the chromosome, as it might lead to a larger structural rearrangement (ring chromosome, chromosomal translocation, etc.) Similarly, a gain of copy can be an interstitial duplication but may also result from an unbalanced chromosomal translocation or a supernumerary chromosome (e.g., Prader–Willi–Angelman duplication/maternal 15q duplication syndrome [49]) (case 18). Therefore, the discussion of molecular CNV findings should include experienced cytogeneticists.

## 5. Conclusions

The NGS-based CNV pipeline allowed us to efficiently detect pathogenic CNVs based on raw NGS data. It (1) improved the overall diagnostic rate of genetic disorders by 5.9% in the population studied, including different disease entities, (2) removed the need for separate microarray-based chromosomal and MLPA analyses in some patients, (3) uncovered new CNVs that would not be detected by the aforementioned techniques, and (4) detected CNVs in patients for which microarray-based chromosomal or MLPA analyses are not the recommended genetic tests based on the clinical indication. However, one of the current limitations is the need for sequencing that is both uniform and with high coverage of genes, particularly for the first exon of genes. In its diagnostic application, ES-based CNV detection may exert its best efficacy when applied to the whole genome rather than to a restricted gene panel. It may also profit from periodic reanalysis of negative cases.

## Figures and Tables

**Table 1:** List of patients with likely pathogenic and pathogenic CNVs detected by the NGS-based CNV pipeline.

Case (Phase)	Disease Category	Clinical Description	NGS Library	NGS CNV Coordinates (Grch37) + Size + Gene If Small CNV	CNV Validation	Acgh/MLPA CNV Results (Grch37)	Known Syndrome (+ Additional Comment)
Case 1 (2)	NDD	DD, mild ID	ES	chr22:44489809-51220722 x1 >6.7 Mb	aCGH 180k	22q13.31q13.33(44481506_51186249)x1	Phelan-McDermid syndrome (qTer)
Case 2 (2)	NDD	DD, language delay, hypotonia	ES	chr15:23609491-28632839 x3 >5 Mb	aCGH 180k	15q11.2q13.1(22765628_28535051)x3	15q11.2-q13.2 microduplication syndrome
Case 3 (2)	BID	immunodeficiency and ID	ES	chr13:103298645-103301836 x0 >4.4 Kb–*TPP2* (exons 20–22)	WGS	Not enough aCGH targets No MLPA available	(patient presented in [24])
Case 4 (2)	NDD	DD, renale acidosis, SS, optic atrophy	ES	chr14:99640489-106236323 x3 >7 Mb	aCGH 180k	14q32.2q32.33(99634561_107278770)x3	14q distal duplications (qTer)
Case 5 (2)	Renal	Renal kystes and DD	ES	chrX: 107224311-107979574 x0 >755 kb	aCGH 180k	Xq22.3(107182490_108105721)x0	
Case 6 (2)	NDD	DD with pyramidal signs & facial dysmorphism	ES	chr2:237028837-242815426 x1 >5 Mb	aCGH 180k	2q37.2q37.3(236980552_243041364)x1	2q37 deletion syndrome (qTer)
Case 7 (2)	NG	Ataxia	ES	chr10:125769666-128860040 x3 chr10:133747956-135379033 x1 >7 Mb	aCGH 180k	10q26.13q26.2(125757754_128852954)x3 10q26.3(130764002_131513932)x3, 10q26.3(131528966_135434178)x1	10q26 deletion syndrome
Case 8 (2)	NDD	Progressive cognitive decline, epilepsy	ES	chr22:18834446-21414817 x1 >2.5 Mb	aCGH 180k	22q11.21(18894835_21464119)x1	22q11.2 microdeletion syndrome
Case 9 (2)	NG	Myoclonic dystonia	ES	chr7:92818581-94540820 x1 >1.7 Mb–including *SGCE*	aCGH 180k	7q21.2q21.3(92776146_94641008)x1	
Case 10 (2)	NDD	Global DD, mild dysmorphic features	ES	chr17:1082962-1657828 x3 >574 Kb	aCGH 180k	17p13.3(1071072_1658551)x3	17p13.3 microduplication syndrome
Case 11 (2)	NDD	DD, ASD	ES	chr1:146461120-147416212 x3 >955 Kb	aCGH 180k	1q21.1q21.2(146542843-149243967)x3	
Case 12 (3)	Hearing loss	Deafness	TsoE	chr15:43892159-43901532 x1 >9.4 Kb–*STRC* (exons 16–28)	MLPA	*STRC* (exon 19-3′UTR) Not enough aCGH targets	+ *STRC* c.4552G>A (p.Gly1518Ser)
Case 13 (3)	NG	Neuroacanthocytosis	ES	chr9:79827886-79828230 x1 >344 bp*–VPS13A* (exons 8–9) chr9:80018153-80018237x1 >84 bp–*VPS13A* (exon 69)	Sanger	Not enough aCGH targets No MLPA available	
Case 14 (3)	Hearing loss	Deafness, neutropenia	TsoE	chr3:69928286-69988332 x1 >60 Kb–*MITF* (exons 1–3)	aCGH 1M	3p13(69917276_69989173)x1 (*MITF*)	
Case 15 (3)	Metabolic disease	Hypophosphatemic rickets	TsoE	chrX:21958944-22151741 x1 >192 Kb–*PHEX*	aCGH 180k	Xp22.11(21950459_22180647)x1 (*PHEX*)	
Case 16 (3)	NDD	Early onset epilepsy	TsoE	chrX:99551276_99663595 x1 >112 Kb–*PCDH19*	MLPA	*PCDH19* fully deleted x1	
Case 17 (3)	NG	Familial spastic paraparesia	TsoE	chr2:32352018_32353548 x1 >1.53 Kb–*SPAST* (exons 8–9)	MLPA	*SPAST* (exons 8–9) Not enough aCGH targets	
Case 18 (3)	NDD	DD, epilepsy, hypotonia, hair anomalies	ES	chr15:22742397-28772634 x4 >6 Mb	aCGH 180k	15q11.1q13.1(20102541_28535051)x4	maternal 15q duplication syndrome, (supernumerary chromosome)

ASD: autism spectrum disorder; bp: base pair; BID: blood-immune disease; DD: developmental delay; ES: exome sequencing; ID: intellectual disability; Kb: kilobase pair; Mb: megabase pair; NDD: neurodevelopmental disorder; NG: neurodegeneration; SS: short stature; TsoE: TruSightOne expanded.

**Table 2:** List of cases with CNVs not detected by the NGS-based CNV pipeline.

Case (Phase)	Disease Category	Clinical Description	NGS Library	CNV Coordinates from NGS (Grch37)	CNV Validation	Acgh CNV Results (Grch37) Size + Gene of Interest	Reason CNV Missed by the CNV Pipeline
Case 19 (2)	NDD	Lissencephaly	ES	Not detected	aCGH 180k	17p13.3(2433963_2548152)x1 >114 kb*–PAFAH1B1* (exons 1–2)	*PAFAH1B1*: exon1 is not targeted by the ES library, exon 2 is targeted with low coverage (Figure S2).
Case 20 (2)	NDD	DD, Epilepsy	ES	chr13:35615071-35697711 x1	aCGH 180k	13q13.3(35522735_35696113)x1 >173 kb–*NBEA* (exons 2–17)	Gene not present in the gene panel analysed originally.
Case 21 (2)	NDD	DD, Epilepsy	ES	chr14:105814831-106370569 x1	aCGH 180k	14q32.33(105807673_107278770)x1 >1.4 Mb–including *PACS2*	Gene not present in the gene panel analysed originally

DD: developmental delay; ES: exome sequencing; kb: kilobase pair; Mb: megabase pair; NDD: Neurodevelopmental disorder.

## Data Availability

The genetic data supporting the findings of this study contain information that could compromise the privacy and/or consent of the participants. We, therefore, provide only the causative CNV variants’ details in the main manuscript tables and/or Supplementary Tables S1–S4.

## Supplementary Materials

The following are available online at https://www.mdpi.com/article/10.3390/genes12091427/s1, Figure S1: Study design and patients enrolled in each phase, Figure S2: Box plots of raw read counts per exon (1–11) of *PAFAH1B1* gene (NM_000430.4, GRCh37). The red dots represent the number of read counts in case 19, while grey dots represent the read-count value of samples from the same batch of 32 individuals, Table S1: Details of the CNV annotations in the pipeline, Table S2: List of patients from Phase 1, to parametrise the NGS-based CNV pipeline, Table S3: Details of the CNVs discovered by the ES-based CNV pipeline, Table S4: CNVs classified as VUS.

## References

[1] Boycott K.M., Rath A., Chong J., Hartley T., Alkuraya F.S., Baynam G., Brookes A.J., Brudno M., Carracedo A., Dunnen J.T.D. (2017). International Cooperation to Enable the Diagnosis of All Rare Genetic Diseases. Am. J. Hum. Genet..

[2] Boycott K.M., Hartley T., Biesecker L.G., Gibbs R.A., Innes A.M., Riess O., Belmont J., Dunwoodie S.L., Jojic N., Lassmann T. (2019). A Diagnosis for All Rare Genetic Diseases: The Horizon and the Next Frontiers. Cell.

[3] Hartley T., Lemire G., Kernohan K.D., Howley H.E., Adams D.R., Boycott K.M. (2020). New Diagnostic Approaches for Undiagnosed Rare Genetic Diseases. Annu. Rev. Genom. Hum. Genet..

[4] McAllister M., Davies L., Payne K., Nicholls S., Donnai D., MacLeod R. (2007). The emotional effects of genetic diseases: Implications for clinical genetics. Am. J. Med Genet. Part A.

[5] Boone P., Bacino C.A., Shaw C., Eng P.A., Hixson P.M., Pursley A.N., Kang S.-H.L., Yang Y., Wiszniewska J., Nowakowska B.A. (2010). Detection of clinically relevant exonic copy-number changes by array CGH. Hum. Mutat..

[6] Sahajpal N., Barseghyan H., Kolhe R., Hastie A., Chaubey A. (2021). Optical Genome Mapping as a Next-Generation Cytogenomic Tool for Detection of Structural and Copy Number Variations for Prenatal Genomic Analyses. Genes.

[7] Neveling K., Mantere T., Vermeulen S., Oorsprong M., van Beek R., Kater-Baats E., Pauper M., van der Zande G., Smeets D., Weghuis D.O. (2021). Next-generation cytogenetics: Comprehensive assessment of 52 hematological malignancy genomes by optical genome mapping. Am. J. Hum. Genet..

[8] Vinkšel M., Writzl K., Maver A., Peterlin B. (2021). Improving diagnostics of rare genetic diseases with NGS approaches. J. Community Genet..

[9] Monroe G.R., Frederix G.W., Savelberg S.M.C., De Vries T.I., Duran K.J., Van Der Smagt J.J., Terhal P.A., Van Hasselt P.M., Kroes H.Y., Verhoeven-Duif N.M. (2016). Effectiveness of whole-exome sequencing and costs of the traditional diagnostic trajectory in children with intellectual disability. Genet. Med..

[10] Lindstrand A., Eisfeldt J., Pettersson M., Carvalho C.M.B., Kvarnung M., Grigelioniene G., Anderlid B.-M., Bjerin O., Gustavsson P., Hammarsjö A. (2019). From cytogenetics to cytogenomics: Whole-genome sequencing as a first-line test comprehensively captures the diverse spectrum of disease-causing genetic variation underlying intellectual disability. Genome Med..

[11] Zhao M., Wang Q., Wang Q., Jia P., Zhao Z. (2013). Computational tools for copy number variation (CNV) detection using next-generation sequencing data: Features and perspectives. BMC Bioinform..

[12] Retterer K., Scuffins J., Schmidt D., Lewis R., Pineda-Alvarez D., Stafford A., Schmidt L., Warren S., Gibellini F., Kondakova A. (2015). Assessing copy number from exome sequencing and exome array CGH based on CNV spectrum in a large clinical cohort. Genet. Med..

[13] Marchuk D.S., Crooks K., Strande N., Kaiser-Rogers K., Milko L.V., Brandt A., Arreola A., Tilley C.R., Bizon C., Vora N.L. (2018). Increasing the diagnostic yield of exome sequencing by copy number variant analysis. PLoS ONE.

[14] Kadalayil L., Rafiq S., Rose-Zerilli M., Pengelly R., Parker H., Oscier D., Strefford P.J.C., Tapper W.J., Gibson J., Ennis S. (2015). Exome sequence read depth methods for identifying copy number changes. Briefings Bioinform..

[15] Plagnol V., Curtis J., Epstein M., Mok K., Stebbings E., Grigoriadou S., Wood N., Hambleton S., Burns S., Thrasher A. (2012). A robust model for read count data in exome sequencing experiments and implications for copy number variant calling. Bioinformormatics.

[16] Royer-Bertrand B., Tsouni P., Mullen P., Xavier B.C., Crettol L.M., Lobrinus A.J., Ghika J., Baumgartner M.R., Rivolta C., Superti-Furga A. (2019). Peripheral neuropathy and cognitive impairment associated with a novel monoallelic HARS variant. Ann. Clin. Transl. Neurol..

[17] Yang H., Wang K. (2015). Genomic variant annotation and prioritization with ANNOVAR and wANNOVAR. Nat. Protoc..

[18] Richards S., Aziz N., Bale S., Bick D., Das S., Gastier-Foster J., Grody W.W., Hegde M., Lyon E., Spector E. (2015). Standards and guidelines for the interpretation of sequence variants: A joint consensus recommendation of the American College of Medical Genetics and Genomics and the Association for Molecular Pathology. Genet. Med..

[19] Quinodoz M., Royer-Bertrand B., Cisarova K., Di Gioia S.A., Superti-Furga A., Rivolta C. (2017). DOMINO: Using Machine Learning to Predict Genes Associated with Dominant Disorders. Am. J. Hum. Genet..

[20] Karczewski K.J., Francioli L.C., Tiao G., Cummings B.B., Alföldi J., Wang Q., Collins R.L., Laricchia K.M., Ganna A., Birnbaum D.P. (2020). The mutational constraint spectrum quantified from variation in 141,456 humans. Nature.

[21] Firth H.V., Richards S.M., Bevan P., Clayton S., Corpas M., Rajan D., Van Vooren S., Moreau Y., Pettett R.M., Carter N.P. (2009). DECIPHER: Database of Chromosomal Imbalance and Phenotype in Humans Using Ensembl Resources. Am. J. Hum. Genet..

[22] Nowakowska B. (2017). Clinical interpretation of copy number variants in the human genome. J. Appl. Genet..

[23] Riggs E.R., Andersen E.F., Cherry A.M., Kantarci S., Kearney H., Patel A., Raca G., Ritter D.I., South S.T., Thorland E.C. (2020). Technical standards for the interpretation and reporting of constitutional copy-number variants: A joint consensus recommendation of the American College of Medical Genetics and Genomics (ACMG) and the Clinical Genome Resource (ClinGen). Genet. Med..

[24] Atallah I., Quinodoz M., Campos-Xavier B., Peter V.G., Fouriki A., Bonvin C., Bottani A., Kumps C., Angelini F., Enders F.B. (2021). Immune deficiency, autoimmune disease and intellectual disability: A pleiotropic disorder caused by biallelic variants in the TPP2 gene. Clin. Genet..

[25] Mandelker D., Amr S., Rehm H.L., Funke B.H., Pugh T., Gowrisankar S., Shakhbatyan R., Duffy E., Bowser M., Harrison B. (2014). Comprehensive Diagnostic Testing for Stereocilin: An approach for analyzing medically important genes with high homology. J. Mol. Diagn..

[26] Dolcetti A., Silversides C.K., Marshall C.R., Lionel A.C., Stavropoulos D.J., Scherer S.W., Bassett A.S. (2012). 1q21.1 Microduplication expression in adults. Genet. Med..

[27] Xavier J., Zhou B., Bilan F., Zhang X., Gilbert-Dussardier B., Viaux-Savelon S., Pattni R., Ho S., Cohen D., Levinson D.F. (2018). 1q21.1 microduplication: Large verbal–nonverbal performance discrepancy and ddPCR assays of HYDIN/HYDIN2 copy number. npj Genom. Med..

[28] Benítez-Burraco A., Barcos-Martínez M., Espejo-Portero I., Fernández-Urquiza M., Torres-Ruiz R., Rodriguez-Perales S., Romero M.S.J. (2018). Narrowing the Genetic Causes of Language Dysfunction in the 1q21.1 Microduplication Syndrome. Front. Pediatr..

[29] Haverfield E.V., Whited A.J., Petras K.S., Dobyns W.B., Das S. (2008). Intragenic deletions and duplications of the LIS1 and DCX genes: A major disease-causing mechanism in lissencephaly and subcortical band heterotopia. Eur. J. Hum. Genet..

[30] Mulhern M.S., Stumpel C., Stong N., Brunner H.G., Bier L., Lippa N., Riviello J., Rouhl R.P.W., Kempers M., Pfundt R. (2018). NBEA: Developmental disease gene with early generalized epilepsy phenotypes. Ann. Neurol..

[31] Olson H.E., Jean-Marçais N., Yang E., Heron D., Tatton-Brown K., Van Der Zwaag P.A., Bijlsma E.K., Krock B.L., Backer E., Kamsteeg E.-J. (2018). A Recurrent De Novo PACS2 Heterozygous Missense Variant Causes Neonatal-Onset Developmental Epileptic Encephalopathy, Facial Dysmorphism, and Cerebellar Dysgenesis. Am. J. Hum. Genet..

[32] Carter N.P. (2007). Methods and strategies for analyzing copy number variation using DNA microarrays. Nat. Genet..

[33] Yu G., Qian X., Wu Y., Li X., Chen J., Xu J., Qi J. (2015). Analysis of gene mutations in PKD1/PKD2 by multiplex ligation-dependent probe amplification: Some new findings. Ren. Fail..

[34] Vona B., Hofrichter M., Neuner C., Schröder J., Gehrig A., Hennermann J.B., Kraus F.B., Shehata-Dieler W., Klopocki E., Nanda I. (2015). DFNB16 is a frequent cause of congenital hearing impairment: Implementation of STRC mutation analysis in routine diagnostics. Clin. Genet..

[35] Plevova P., Paprskarova M., Tvrda P., Turska P., Slavkovsky R., Mrazkova E. (2017). STRC Deletion is a Frequent Cause of Slight to Moderate Congenital Hearing Impairment in the Czech Republic. Otol. Neurotol..

[36] Moteki H., Azaiez H., Sloan-Heggen C.M., Booth K.T., Nishio S.-Y., Wakui K., Yamaguchi T., Kolbe D.L., Iwasa Y.-I., Shearer A. (2016). Detection and Confirmation of Deafness-Causing Copy Number Variations in the STRC Gene by Massively Parallel Sequencing and Comparative Genomic Hybridization. Ann. Otol. Rhinol. Laryngol..

[37] Kucharík M., Budiš J., Hýblová M., Minárik G., Szemes T. (2021). Copy Number Variant Detection with Low-Coverage Whole-Genome Sequencing Represents a Viable Alternative to the Conventional Array-CGH. Diagnostics.

[38] Mandelker D., Schmidt R.J., Ankala A., Gibson K.M., Bowser M., Sharma H., Duffy E., Hegde M., Santani A., Lebo M. (2016). Navigating highly homologous genes in a molecular diagnostic setting: A resource for clinical next-generation sequencing. Genet. Med..

[39] Yao R., Zhang C., Yu T., Li N., Hu X., Wang X., Wang J., Shen Y. (2017). Evaluation of three read-depth based CNV detection tools using whole-exome sequencing data. Mol. Cytogenet..

[40] Kim H.-Y., Choi J.-W., Lee J.-Y., Kong G. (2017). Gene-based comparative analysis of tools for estimating copy number alterations using whole-exome sequencing data. Oncotarget.

[41] Du C., Pusey B.N., Adams C.J., Lau C.C., Bone W.P., Gahl W.A., Markello T.C., Adams D.R. (2016). Explorations to improve the completeness of exome sequencing. BMC Med. Genom..

[42] Zhou J., Zhang M., Li X., Wang Z., Pan D., Shi Y. (2021). Performance comparison of four types of target enrichment baits for exome DNA sequencing. Hereditas.

[43] Bamshad M.J., Nickerson D.A., Chong J.X. (2019). Mendelian Gene Discovery: Fast and Furious with No End in Sight. Am. J. Hum. Genet..

[44] Posey J.E., Genomics C.F.M., O’Donnell-Luria A.H., Chong J.X., Harel T., Jhangiani S.N., Akdemir Z.H.C., Buyske S., Pehlivan D., Carvalho C.M.B. (2019). Insights into genetics, human biology and disease gleaned from family based genomic studies. Genet. Med..

[45] Wenger A.M., Guturu H., Bernstein J.A., Bejerano G. (2016). Systematic reanalysis of clinical exome data yields additional diagnoses: Implications for providers. Genet. Med..

[46] Basel-Salmon L., Orenstein N., Markus-Bustani K., Ruhrman-Shahar N., Kilim Y., Magal N., Hubshman M.W., Bazak L. (2018). Improved diagnostics by exome sequencing following raw data reevaluation by clinical geneticists involved in the medical care of the individuals tested. Genet. Med..

[47] Fung J.L.F., Yu M.H.C., Huang S., Chung C.C.Y., Chan M.C.Y., Pajusalu S., Mak C.C.Y., Hui V.C.C., Tsang M.H.Y., Yeung K.S. (2020). A three-year follow-up study evaluating clinical utility of exome sequencing and diagnostic potential of reanalysis. npj Genom. Med..

[48] Tan N.B., Stapleton R., Stark Z., Delatycki M.B., Yeung A., Hunter M.F., Amor D.J., Brown N.J., Stutterd C.A., McGillivray G. (2020). Evaluating systematic reanalysis of clinical genomic data in rare disease from single center experience and literature review. Mol. Genet. Genom. Med..

[49] Kalsner L., Chamberlain S.J. (2015). Prader-Willi, Angelman, and 15q11-q13 Duplication Syndromes. Pediatr. Clin. N. Am..

