# Supplementary material for: CNV Detection from Exome Sequencing Data in Routine Diagnostics of Rare Genetic Disorders: Opportunities and Limitations
Source: Genes (Basel). 2021 Sep 16;12(9):1427. doi: 10.3390/genes12091427 (PMC8472439; doi:10.3390/genes12091427)
Supplement: Supplementary file 1 [file genes-12-01427-s001.zip › Figure_S1_Royer-Bertrand.pdf]

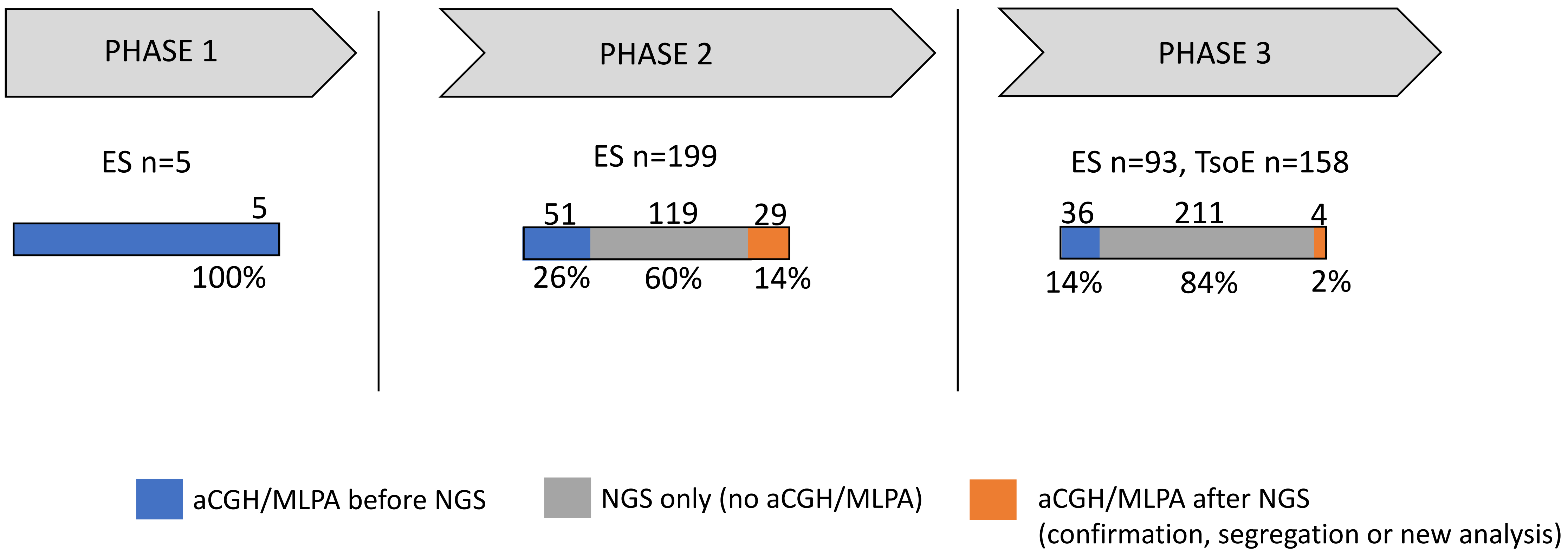

Figure S1 : Study design and patients enrolled in each phase. Number of NGS patients in each phase, and which sequencing methods were used for each of them. *ES: Exome Sequencing; TsoE: TruSightOne Expended.*
