# Supplementary material for: CNV Detection from Exome Sequencing Data in Routine Diagnostics of Rare Genetic Disorders: Opportunities and Limitations
Source: Genes (Basel). 2021 Sep 16;12(9):1427. doi: 10.3390/genes12091427 (PMC8472439; doi:10.3390/genes12091427)
Supplement: Supplementary file 1 [file genes-12-01427-s001.zip › Table_S1_Royer-Bertrand_Revision.pdf]

Type	Annotation	Details
CNV	Localisation	Genomic coordinates of the CNV (GRCh37)<br>+ Cytoband (GRCh37)
	Minimal size of the CNV	In base pair
	Minimal number of exons impacted	Equivalent to the number of targets inside the CNV boundaries in the sequencing library
	Ploidy and quality, via the reads' ratio	Ratio of observed reads in the CNV versus average reads of the reference:<br>- ratio= 1 : 2 normal copies,<br>- +/- 0.5 : loss of 1 copy, +/- 0 : loss of 2 copies<br>- +/- 1.5 : gain of 1 copy, +/- 1.8 : gain of 2 copies
	Frequency	- in the batch under process<br>- in the internal database of NGS patients (ES SSV7 and TsoE)<br>- in control database (Konrad), provided by ExomeDepth software
	Known pathogenic CNVs	Extracted from the OMIM database, for large CNVs. CNVs detected in our patients might be inside those known pathogenic CNVs, overlapping with them or bigger than them.
	180K and 1M aCGH targets	Number of targets from 180K and 1M aCGH, inside the CNV boundaries.
	pTer and qTer flag	Flag CNVs close to the pTer and qTer regions of the chromosomes, by calculating the number of available library targets before (for pTer) and after (for qTer) the CNV.
Genes	RefGenes	Genes names from RefGenes present in the CNV
	OMIM	Disease description of genes present in the CNV
	Domino	Domino score (Dominant/Recessif) of genes present in the CNV
	Happloinsufficiency/ Triplosensitivity score	ClinGen Dosage Sensitivity Map (https://dosage.clinicalgenome.org/)

Table S1: Details of the CNV annotations in the pipeline.
