# Supplementary material for: CNV Detection from Exome Sequencing Data in Routine Diagnostics of Rare Genetic Disorders: Opportunities and Limitations
Source: Genes (Basel). 2021 Sep 16;12(9):1427. doi: 10.3390/genes12091427 (PMC8472439; doi:10.3390/genes12091427)
Supplement: Supplementary file 1 [file genes-12-01427-s001.zip › Table_S2_Royer-Bertrand_Revision.pdf]

Case	Disease Category	Original technique	CNV results (GRCh37) aCGH /MLPA size + gene of interest	CNV coordinates from NGS (GRCh37)
**Case A**	NDD	180k aCGH	22q13.33(51123491-51178264) x1<br>55kb – including *SHANK3*	chr22:51123014-51220722 x1<br>>97kb – including *SHANK3*
**Case B**	NG	MLPA PMP22	14q11.2(20002226-20404761) x1<br>106bp – *PMP22* (exon 5)	chr14:20002226-20404761 x1<br>106bp – *PMP22* (exon 5)
**Case C**	NDD	180k aCGH	16q11.2(28824794-29042118) x1<br>217kb – including *SH2B1*	chr16:28834582-29064047 x1<br>>229kb – including *SH2B1*
**Case D**	NDD	180k aCGH	Xp11.22(52954520-53742476) x3<br>>788kb – including *HUWE1*	chrX:53105805-53681051 x3<br>>575kb - including *HUWE1*
**Case E**	NDD	180k aCGH	22q11.21(18894835-21801661) x3<br>>2.9Mb	chr22:18893889-21481925 x3<br>>2.58Mb

Kb: Kilobase pair; Mb: Megabase pair; NDD: Neurodevelopmental disorder; NG: Neurodegeneration.

Table S2: List of patients from Phase 1, to parametrize the NGS-based CNV pipeline.
