# Supplementary material for: CNV Detection from Exome Sequencing Data in Routine Diagnostics of Rare Genetic Disorders: Opportunities and Limitations
Source: Genes (Basel). 2021 Sep 16;12(9):1427. doi: 10.3390/genes12091427 (PMC8472439; doi:10.3390/genes12091427)
Supplement: Supplementary file 1 [file genes-12-01427-s001.zip › Table_S3_Royer-Bertrand_Revision_version2.pdf]

Case (Phase)	CNV coordinates of the largest boundaries detected (GRCh37)	cytoband	Minimal size of the CNV	Classification	Criteria	Genes in the CNV (mim morbid genes underlined)	Gene(s) responsible for the phenotype
**Case 1 (2)**	chr22: 44481506-51220722 x1	22q13.31q13.33	>6.7Mb	Pathogenic	Known syndrome (MIM: #606232)	*PARVB,PARVG,KIAA1644,,LDOC1L,PRR5,PRR5-ARHGAP8, ARHGAP8, PHF21B, NUP50, KIAA0930,UPK3A,FAM118A,SMC1B,RIBC2,FBLN1, ATXN10,WNT7B,C22orf26,PPARA,CDPF1,PKDREJ, TTC38,GTSE1,TRMU,CELSR1,GRAMD4,CERK,TBC1D22A,FAM19A5,C22orf34,BRD1,ZBED4,ALG12,CRELD2,PIM3,IL17REL,TTLL8,MLC1,MOV10L1,PANX2, TRABD,TUBGCP6,HDAC10,MAPK12,MAPK11,PLXNB2,DENND6B,PPP6R2,SBF1,ADM2,MIOX,LMF2,NCAPH2,,SCO2,TYMP,ODF3B,KLHDC7B,SYCE3,CPT1B,CHKB-CPT1B, CHKB, MAPK8IP2, ARSA,SHANK3,ACR,RABL2B*	*SHANK3*
**Case 2 (2)**	chr15: 22765628-28632839 x3	15q11.2q13.1	>5.8Mb	Pathogenic	Known syndrome (MIM: #608636)	*TUBGCP5,CYFIP1,NIPA2,NIPA1,GOLGA8I,,GOLGA8S,GOLGA6L2,MKRN3,MAGEL2,NDN,NPAP1,SNRPN,SNURF,UBE3A,ATP10A,GABRB3,,GABRA5,GABRG3,,OCA2,HERC2,GOLGA8F*	PWACR region (BP1-BP3)
**Case 3 (2)**	chr13:103298645-103301836 x0	13q33.1	>4.4Kb	Pathogenic	See article [31]	*TPP2*	*TPP2*
**Case 4 (2)**	chr14: 99634561-107278770 x3	14q32.2q32.33	>7.6Mb	Likely pathogenic	Dozen of patients with similar duplication (ORPHA: 1705), large CNV, absent from control	*BCL11B,SETD3,CCNK,CCDC85C,HHIPL1,CYP46A1, EML1,EVL,DEGS2,YY1,SLC25A29,SLC25A47,WARS, WDR25,BEGAIN,DLK1,RTL1,DIO3,PPP2R5C, DYNC1H1, HSP90AA1, WDR20, MOK,ZNF839,CINP,TECPR2,ANKRD9,RCOR1,TRAF3,AMN,CDC42BPB,EXOC3L4,TNFAIP2,EIF5,MARK3,CKB,TRMT61A,BAG5,KLC1,APOPT1,XRCC3,ZFYVE21,PPP1R13B,C14orf2,TDRD9,RD3L,ASPG,KIF26A,C14orf144,TMEM179,C14orf180,INF2, ADSSL1,SIVA1, AKT1, ZBTB42, CEP170B, PLD4, AHNAK2, C14orf79,CDCA4,GPR132,JAG2,NUDT14,BRF1,BTBD6,PACS2,TEX22,MTA1,CRIP2,CRIP1,C14orf80,TMEM121,KIAA0125*	14q32.2 locus (*DKL1/RTL1*)
**Case 5 (2)**	chrX: 107182490-108105721 x0	Xq22.3	>900kb	Pathogenic	Haploinsufficiency of *COL4A5* (ISCA-11460)	*TEX13B,VSIG1,PSMD10,ATG4A,COL4A6,COL4A5,,IRS4*	*COL4A5, IRS4*

**Case 6 (2)**	chr2: 236980552-243041364 x1	2q37.2q37.3	>6Mb	Pathogenic	Known syndrome (MIM: #600430)	*AGAP1,GBX2,ASB18,IQCA1,ACKR3,COPS8,,COL6A3,MLPH,PRLH,RAB17,LRRFIP1,RBM44,RAMP1,UBE2F,UBE2F-SCLY, SCLY, ESPNL,KLHL30,FAM132B,ILKAP,HES6,PER2,TRAF3IP1,ASB1,TWIST2,HDAC4,NDUFA10,OR6B2,PRR21,OR6B3,MYEOV2,OTOS,GPC1,ANKMY1,DUSP28,RNPEPL1,CAPN10,GPR35,AQP12B,AQP12A,KIF1A,AGXT,C2orf54,SNED1,MTERFD2,PASK,PPP1R7,ANO7, HDLBP,SEPT2,FARP2,STK25,BOK,THAP4,ATG4B,DTYMK,ING5,D2HGDH,GAL3ST2,NEU4,PDCD1,CXXC111*	*HDAC4* is contributing, but not sufficient to cause the phenotype. Other genes are unknown
**Case 7 (2)**	chr10: 125757754-128860040 x3; chr10: 130764002_131513932 x3; chr10: 131528966-135434178 x1	10q26.13q26.2; 10q26.3; 10q26.3	>3.1 Mb; 750kb; >3.9Mb	Pathogenic	Known syndrome (MIM: #609625)	*CHST15,OAT,NKX1-2,LHPP,,FAM53B,METTL10,FAM175B,ZRANB1,CTBP2,TEX36,EDRF1,MMP21,UROS,BCCIP,DHX32,FANK1,ADAM12,C10orf90,DOCK,MGMT,EBF3,GLRX3, TCERG1L,PPP2R2D,BNIP3,JAKMIP3,DPYSL4,STK32C,LRRC27,PWWP2B,C10orf91,INPP5A,NKX6-2,TTC40,GPR123,KNDC1,UTF1,VENTX,ADAM8,TUBGCP2,ZNF511,CALY,PRAP1,FUOM,ECHS1,PAOX,MTG1,SPRN,CYP2E1,SYCE1*	*EBF3*
**Case 8 (2)**	chr22:18834446-21464119 x1	22q11.21	>2.6Mb	Pathogenic	Known syndrome (MIM: #192430)	*DGCR6,PRODH,DGCR2,DGCR14,TSSK2,GSC2,SLC25A1,CLTCL1,HIRA,C22orf39,MRPL40,UFD1L,CDC45,CLDN5,SEPT5,GP1BB,TBX1,GNB1L,C22orf29,TXNRD2,COMT, ARVCF,TANGO2,DGCR8,TRMT2A,RANBP1,ZDHHC8,RTN4R,DGCR6L,GGTLC3,RIMBP3,FAM230A,USP41,ZNF74,SCARF2,KLHL22,MED15,PI4KA,SERPIND1,SNAP29,CRKL,AIFM3,LZTR1,THAP7,P2RX6,SLC7A4*	*TBX1*
**Case 9 (2)**	chr7: 92776146-94641008 x1	7q21.2q21.3	>1.8Mb	Pathogenic	Known deletion, *SGCE* GeneReviews, 2020	*AMD9L,HEPACAM2,CCDC132,CALCR,GNGT1,TFPI2,GNG11,BET1,COL1A2,CASD1,SGCE,PEG10,PPP1R9A*	*SGCE*
**Case 10 (2)**	chr17: 1071072-1658551 x3	17p13.3	>580Kb	Pathogenic	Known syndrome (MIM: #613215)	*ABR,BHLHA9,TUSC5,YWHAE,CRK,MYO1C,INPP5K, PITPNA,SLC43A2,SCARF1,RILP,PRPF8,TLCD2,WDR81,SERPINF2*	*YWHAE*

**Case 11 (2)**	chr1: 146461120-149243967 x3	1q21.1q21.2	>2.7Mb	Pathogenic	Known syndrome (MIM #612475)	*NBPF12,PRKAB2,FMO5,CHD1L,BCL9,ACP6,GJA5, GJA8,GPR89B,NBPF24,,PPIAL4A,NBPF14,PPIAL4D ,NBPF20,NBPF15,NBPF16*	None known but *CHD1L, ACP6, TL3*, and *ROBO1* were labeled as candidates [27]
**Case 12 (3)**	chr15:43892159-43901532 x1	15q15.3	>9.4Kb	Pathogenic	Gene known to have partial deletion (biallelic disorder), compound heterozygous with SNV	*CKMT1B,STRC*	*STRC*
**Case 13 (3)**	chr9:79827886-79828230 x1 chr9:80018153-80018237x1	9q21.2	>344bp >84bp	Pathogenic	Gene known to have partial deletion (biallelic disorder), compound heterozygous	*VPS13A*	*VPS13A*
**Case 14 (3)**	chr3: 69917276-69989173 x1	3p13	>70Kb	Pathogenic	Haploinsufficiency of *MITF* (ISCA-4115)	*MITF*	*MITF*
**Case 15 (3)**	chrX: 21950459-22180647 x1	Xp22.11	>230Kb	Pathogenic	Haploinsufficiency of *PHEX* (ISCA-35047), *PHEX* GeneReviews, 2017	*SMS, PHEX*	*PHEX*
**Case 16 (3)**	chrX: 99551276-99663595 x1	Xq22.1	>112Kb	Pathogenic	Haploinsufficiency of *PCDH19* (ISCA-6010), known deletion (HGMD Pro)	*PCDH19*	*PCDH19*
**Case 17 (3)**	chr2:32352018_32353548 x1	2p22.3	>1.53Kb	pathogenic	Haploinsufficiency of *SPAST* (ISCA-16468), *SPAST* GeneReviews, 2019	*SPAST*	*SPAST*
**Case 18 (3)**	chr15: 20102541-28772634 x4	15q11.1q13.1	>8Mb	Pathogenic	Known syndrome, maternal 15q duplication syndrome GeneReviews, 2021	*GOLGA6L6,,POTEB2,POTEB,OR4M2,OR4N4,GOLGA6L1,TUBGCP5,CYFIP1,NIPA2,NIPA1,GOLGA8I,GOLGA8S,GOLGA6L2,MKRN3,MAGEL2,NDN,NPAP1, SNRPN,SNURF,UBE3A,ATP10A,GABRB3,,GABRA5, GABRG3,OCA2,HERC2,GOLGA8F,GOLGA8G*	PWACR region (BP1-BP3)

Table S3: Details of the CNVs discovered by the ES-based CNV pipeline.
bp: base pair; Kb: Kilobase pair; Mb: Megabase pair, PWACR: Prader Willi Angelman Critical Region
