# Supplementary material for: CNV Detection from Exome Sequencing Data in Routine Diagnostics of Rare Genetic Disorders: Opportunities and Limitations
Source: Genes (Basel). 2021 Sep 16;12(9):1427. doi: 10.3390/genes12091427 (PMC8472439; doi:10.3390/genes12091427)
Supplement: Supplementary file 1 [file genes-12-01427-s001.zip › Table_S4_Royer-Bertrand_Revision.pdf]

Case (Phase)	Disease Category	Clinical description	NGS library	NGS CNV coordinates (GRCh37) + size + gene if small CNV	CNV validation	aCGH CNV results (GRCh37)	Comment
**Case 22 (2)**	NG	Ataxia	ES	chr1:161275667-161310445 x3 >34kb – including *MPZ*	aCGH 180k	1q23.3(161275175_161316088)x3 >41kb – including *MPZ*	Same CNV present in the healthy mother
**Case 23 (3)**	Renal disease	Renal malformation	ES	chr12:66709023-66747343 x3 >38kb – including *GRIP1*	no	aCGH not done	+ *GRIP1* c.260G>A, p.Gly87Glu
**Case 24 (2)**	NDD	DD, language delay, obesity	ES	chr1:160765979-161228773x3 >462kb	aCGH 180k	1q23.3(160676568_161291124)x3 >615kb	Mosaicism of the duplication (50-55%)

DD: Developmental Delay; ES: Exome Sequencing; kb: Kilobase pair; Mb: Megabase pair; NDD: Neurodevelopmental disorder; NG: Neurodegeneration.

Table S3: CNVs classified as VUS.
