# Supplementary figures and images for: CNV Detection from Exome Sequencing Data in Routine Diagnostics of Rare Genetic Disorders: Opportunities and Limitations
Source: Genes (Basel). 2021 Sep 16;12(9):1427. doi: 10.3390/genes12091427 (PMC8472439; doi:10.3390/genes12091427)

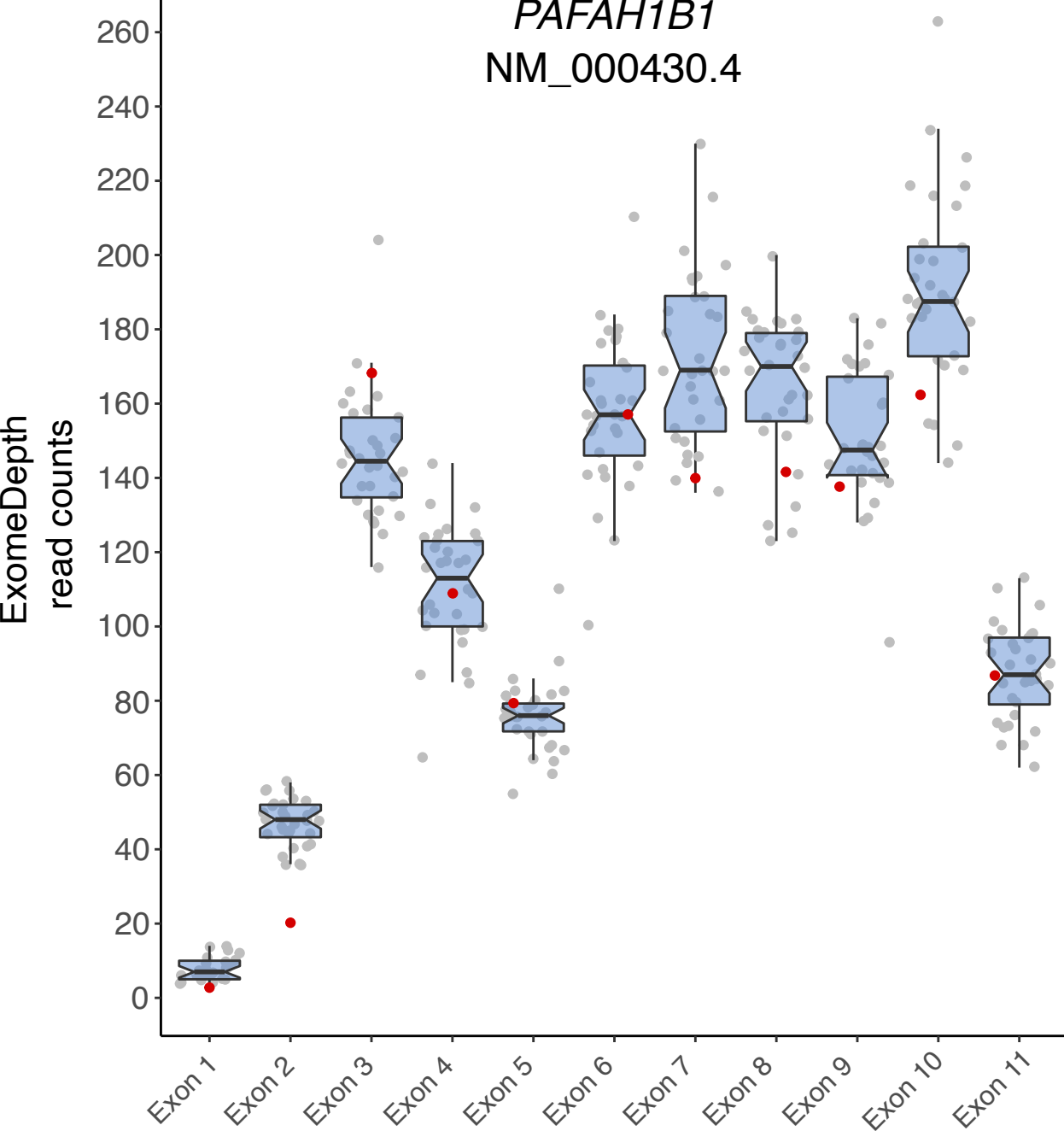

Supplement: Supplementary file 1 [file genes-12-01427-s001.zip › Figure_S2_Royer-Bertrand.pdf]
